# Computational Studies of Au(I) and Au(III) Anticancer MetalLodrugs: A Survey

**DOI:** 10.3390/molecules26247600

**Published:** 2021-12-15

**Authors:** Iogann Tolbatov, Alessandro Marrone, Cecilia Coletti, Nazzareno Re

**Affiliations:** 1 Institut de Chimie Moleculaire de l’Université de Bourgogne (ICMUB), Université de Bourgogne Franche-Comté (UBFC), Avenue Alain Savary 9, 21078 Dijon, France; tolbatov.i@gmail.com; 2 Dipartimento di Farmacia, Università degli Studi “G. D’Annunzio” Chieti-Pescara, Via dei Vestini, 66100 Chieti, Italy; amarrone@unich.it (A.M.); ccoletti@unich.it (C.C.)

**Keywords:** antitumor complexes, Au(I) complexes, Au(III) complexes, anticancer metallodrugs, computations

## Abstract

Owing to the growing hardware capabilities and the enhancing efficacy of computational methodologies, computational chemistry approaches have constantly become more important in the development of novel anticancer metallodrugs. Besides traditional Pt-based drugs, inorganic and organometallic complexes of other transition metals are showing increasing potential in the treatment of cancer. Among them, Au(I)- and Au(III)-based compounds are promising candidates due to the strong affinity of Au(I) cations to cysteine and selenocysteine side chains of the protein residues and to Au(III) complexes being more labile and prone to the reduction to either Au(I) or Au(0) in the physiological milieu. A correct prediction of metal complexes’ properties and of their bonding interactions with potential ligands requires QM computations, usually at the ab initio or DFT level. However, MM, MD, and docking approaches can also give useful information on their binding site on large biomolecular targets, such as proteins or DNA, provided a careful parametrization of the metal force field is employed. In this review, we provide an overview of the recent computational studies of Au(I) and Au(III) antitumor compounds and of their interactions with biomolecular targets, such as sulfur- and selenium-containing enzymes, like glutathione reductases, glutathione peroxidase, glutathione-S-transferase, cysteine protease, thioredoxin reductase and poly (ADP-ribose) polymerase 1.

## 1. Introduction

The earliest recorded use of gold in medicine is traced back to 2500 BC when it was used in ancient China; much later, in seventeenth-century Europe, it was used against melancholy, fainting, fevers, and against syphilis in the late nineteenth century [1]. Gold complexes are ubiquitously utilized for the treatment of severe rheumatoid arthritis [2]. Moreover, the interest in gold(I) and gold(III) complexes in anticancer therapy has been recently growing owing to the strong antitumor activity in vitro and in vivo as well as to the relative chemical stability easily modulated by the addition of various ligands [3,4].

Computational chemistry nowadays plays an important role in the search for new drug candidates due to the increasing efficacy of methods based on quantitative structure-activity relationships [5]. These methods yield reliable statistical models which permit establishing a correlation between chemical properties of complexes and their biological activities, thus giving insights into their therapeutic mode of action [6]. This methodology is indispensable for the development of chemical compounds for medical use since it allows to find relationships between their affinities toward binding to their expected biotargets and their structural properties. This approach is based on scanning the databases of chemical complexes versus the intended targets, such a tactic being predominant for organic compounds. However, it is much less efficient in the case of metal complexes due to a specific activation path usual for all metallodrugs, relying on the ligand substitution by the biotarget and their ability to operate against a plethora of biotargets owing to the promiscuity of metallodrugs [7,8,9]. This results in the widespread usage of ab initio and DFT methods for studies of metal-based scaffolds intended for medical use [10,11,12,13,14].

In this work, we review recent computational studies of Au(I) and Au(III) antitumor compounds, including joint experimental/computational investigations. We consider this topic to be crucial for the design of novel gold-based metallodrugs and for the further development of existing metalloscaffolds for clinical use.

## 2. Computational Studies of Au(I) Metallodrugs

The mechanism of action of Au(I) complexes is not yet elucidated but several investigations have suggested that they may trigger apoptosis via inhibition of selenium- and sulfur-containing enzymes such as glutathione reductases, glutathione peroxidase, glutathione-S-transferase, cysteine protease, thioredoxin reductase (TrxR) and poly (ADP-ribose) polymerase 1 (PARP-1) [2,3,4].

**Figure 1:**
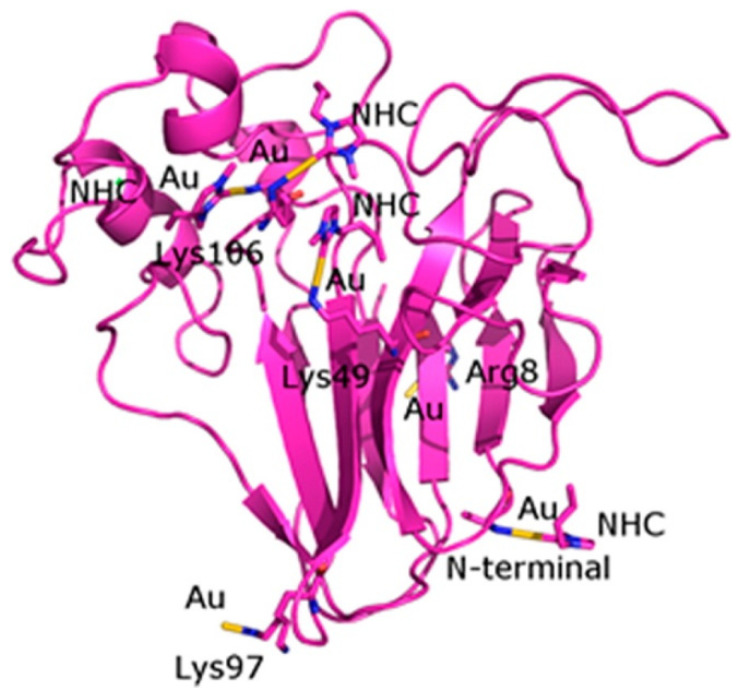
Overall structure of thaumatin-Au(NHC)Cl (with NHC = 1-butyl-3-methyl-imidazole-2-ylidene) (see ref. [19]).

Indeed, metal ion Au(I) is a soft metal center, demonstrating a strongly pronounced affinity toward soft ligands such as thiols of cysteines and thioethers of methionines [15] and even more with selenols of selenocysteine residues [16]. Nevertheless, several X-ray crystallographic studies proved that Au(I) ions are able to bind solvent-exposed His [17,18] even when free thiols are available. Moreover, Au(I) complexes may bind the Arg and Lys side chains as well as the N-terminal of Ala [19], albeit in the absence of available cysteines, methionines, and histidines. For instance, binding of Au(NHC)Cl (with NHC = 1-butyl-3-methyl-imidazole-2-ylidene) to the model protein thaumatin occurs at lysine side chains and at the N-terminal tail; the metal binds the protein after releasing Cl^−^ ligand, but retaining NHC fragment (Figure 1) [19]. Linear Au(I) complexes are robust inhibitors of the Se-free enzyme glutathione reductase (GR) due to the high selectivity toward thiols [20,21].

**Figure 2:**
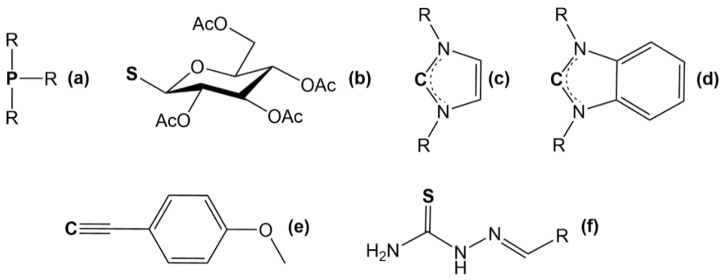
Most common ligands in Au(I) antitumor complexes: phosphine with R = Ph, Me, Et (**a**); thiosugar (**b**); NHC complexes imidazol-2-ylidene (**c**) and benzimidazol-2-ylidene with R = Me, Et (**d**); alkynyl (**e**); thiosemicarbazone (**f**) with R = phenyl, *p*-methoxyphenyl, thiophene, furan, pyridinyl, and *p*-hydroxyphenyl. Atoms directly bound to Au(I) are in bold.

Among the most common Au(I) compounds synthesized and tested as potential anticancer drugs are those bearing phosphine [22], thiosugar [23], N-heterocyclic carbenes (NHC)[24], alkynyl [25], and other sulfur-based ligands such as thiosemicarbazone [26] (Figure 2).

### 2.1. Auranofin and Auranofin Analogs

**Figure 3:**
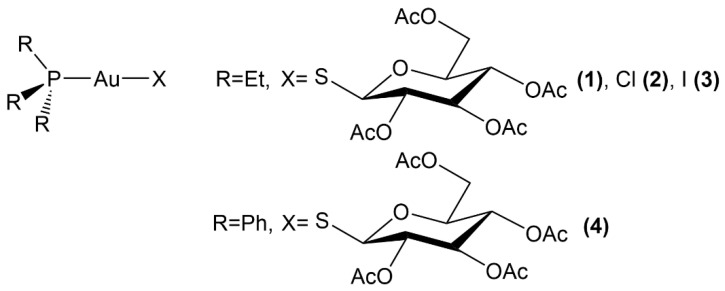
Au(I)-based structures, (**1**) auranofin, (**2**) Et_3_PAuCl, (**3**) auranofin iodide, and (**4**) auranofin with phenyl substituents.

The most prominent gold-based metallodrug is auranofin (2,3,4,6-tetra-O-acetyl-L-thio-β-D-glyco-pyranosato-S-(triethyl-phosphine)-gold(I) (AF) (Figure 3, structure **1**) [23]. Initially known as an anti-arthritic agent, it was later revealed as an antiviral, antibacterial, antiparasitic, and antifungal therapeutics [27,28,29]. Additionally, the antitumoral activity of this agent makes it efficacious in inducing apoptosis in various types of human cancer cells: prostate, ovarian, lung, breast, blood, and bone [30,31]. Its mode of action greatly differs from the platinum-based complexes, based on DNA binding, since it selectively targets sulfur- and selenium-containing proteins. For example, AF extensively and quickly binds albumin [32], proteasome system [33], the NF-κB protein complex [34], and thioredoxin reductase (TrxR) [35], all of which are incorporated in defining the anticancer activity. However, TrxR is believed to be the main target of AF since the gold fragment of AF coordinates tightly to the redox-active site of TrxR, therefore causing oxidative stress and ultimately resulting in cellular apoptosis [36]. However, despite numerous experimental and computational studies, the *modus operandi* of AF is still elusive at the biomolecular level. There is a ubiquitous agreement that the anticancer effect is produced by the [Au(PEt_3_)]^+^ cation whilst the thiosugar operates principally as a carrier ligand [37]. In the last decade, mechanistic consequences of the replacement of thiosugar moiety with different ligands have been studied, and it has been noted that the biological anticancer activity of the [Au(PEt_3_)]^+^ fragment is not affected by this alteration [38].

The reactivity of the [Au(PMe_3_)]^+^ fragment of auranofin with the cysteine, selenocysteine was studied at DFT level of theory, as well as its interaction with the solvent-exposed subunit of TrxR, a tetrapeptide H_2_NGlyCysXGlyCOOH (X = Cys, Sec) which is responsible for the catalysis of the NADPH-dependent reduction of thioredoxins [39]. These calculations displayed a higher acidity of Se-H with respect to S-H, causing a stronger binding of gold at the selenium site compared to sulfur. Moreover, it was shown that the reducing capability of H_2_NGlyCysSecGlyCOOH increases after deprotonation of Sec or diminishes in case of gold coordination at Sec.

To shed light on the mechanism behind the inhibition of TrxR, a computational investigation at DFT level analyzed the ligand exchange reactions of auranofin with the potential target amino acids Cys, Sec, His, and Lys [40]. Both the cleavage of thioglucose and triethylphosphine were studied in aqueous solution at physiological conditions. The thioglucose substitution was established to be kinetically more favorable with the activation barriers in the order Lys < His < Sec < Cys yet the reaction free energies following the trend Sec << Cys<< Lys < His, as expected from the soft nature of gold(I) metal center and the softness of Sec and Cys ligands. The structural analysis of transition states permitted to correlate the reduction of activation enthalpy with the increase in reactive angle, thus envisioning the possibility to modulate auranofin reactivity by adding bulky ligands on the thiosugar moiety.

Another combined NMR and computational investigation at DFT level of the reactions between auranofin and models of thiol and selenol nucleophiles present in TrxR, conducted in chloroform and methanol, revealed important differences related to the polarity of the reaction milieu [41]. It was found that the thiosugar moiety was reversibly substituted by both Cys and Sec, albeit with large differences in the equilibrium constants, being ca. 1 and more than 1000 for the binding at S and Se, respectively. In the polar methanol, the reaction was demonstrated to be more complex, with the phosphine group being able to participate in the ligand exchange. Similar conclusions were made on the reactivity of Et_3_PAuCl (Figure 3, structure **2**).

Computational studies on auranofin and the analogous chlorotriethylphosphine gold complex by means of hybrid density functional theory at the B3LYP/DZVP level [42] revealed metal binding to cysteine and selenocysteine and showed that auranofin could bind selenium in glutathione peroxidase enzymes. These results are corroborated by a mass spectrometry investigation [42], demonstrating that substitution of thiosugar in auranofin with chloride does not alter its reactivity and proving the gold triethylphosphine moiety to be the active ligand.

The iodide analog of auranofin, Au(PEt_3_)I (AF-I), was found to have an exceptionally advantageous biochemical profile demonstrating powerful cytotoxic activity in vitro against several cancer types and an almost full and prompt remission in an orthotopic in vivo mouse model of ovarian cancer (Figure 3, structure **3**) [38]. Furthermore, the lack of severe toxic side effects leads to excellent toleration of AF-I even when high doses are administered. This was the motivation behind a recent combined experimental/theoretical investigation of the mechanistic aspects of the antitumor activity of AF-I, where its reactivity toward molecular models of relevant aminoacidic residues, namely histidine, cysteine, methionine, and selenocysteine, was analyzed, and the enhanced antitumoral activity of AF-I compared to AF was shown [43]. The thermodynamic and kinetic computational results corroborate the conclusion that only Cys and Sec in their anionic forms can favorably substitute thiosugar and iodide in AF and AF-I, respectively, with activation energy for iodide substitution in AF-I being substantially lower [43] than the thiosugar one in AF. This unequivocal preference toward Cys and Sec residues reinforces the understanding that thioredoxin reductase is the most probable target of these gold complexes. Moreover, the marked reactivity of AF-I toward these nucleophile sites makes this Au(I) complex a promising scaffold for the further exploration of its anticancer properties in vivo.

Although auranofin is currently widely studied as an anticancer drug, its original use is the treatment of rheumatoid arthritis [44]. A joint experimental and computational study on the effect of different decoration of the auranofin phosphine ligand towards its in vitro inhibition of cathepsin B, a highly homologous lysosomal cysteine protease participating in the processing of antigens, revealed that sequential replacement of ethyl by phenyl moieties causes a drastic increase in efficacy [45]. It was shown that while AF approaches at cathepsin B by fitting only one hydrophobic site, the resulting Au(I) complexes, endowed with bulky phenyl substituents, may fit two hydrophobic pockets of the enzyme, thus enhancing the binding of this metallodrug at its protein target (Figure 3, structure **4**).

**Figure 4:**
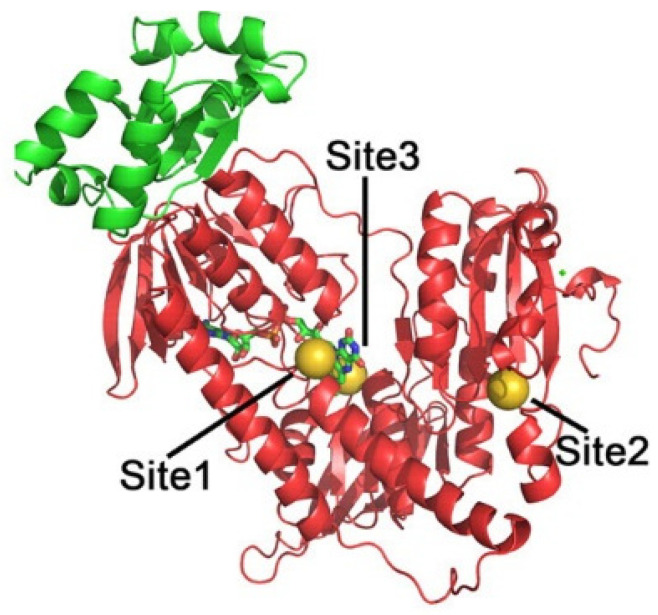
A three-dimensional model of one subunit of *Schistosoma mansoni* thioredoxin-glutathione reductase, SmTGR, is shown with the three gold-binding sites. The glutaredoxin domain of TGR is represented in green, whereas the thioredoxin domain is shown in red, and the bound flavin is also highlighted. (see ref. [46]).

There is a body of experimental evidence on the efficacy of auranofin as an antiparasitic and anti-Schistosoma agent, yet the molecular mechanisms behind its inhibition of *Schistosoma mansoni*, the pathogenic parasite that causes intestinal schistosomiasis, is largely unknown [46]. A possible drug target is the selenoprotein thioredoxin-glutathione reductase (TGR), a key enzyme in the pathway of the parasite for detoxification of reactive oxygen species. The X-ray structure of wild type TGR incubated with AF has shown that the inhibitor is indeed the gold ion released from AF, which is bound at three different sites––mainly Cys residues––not directly involving the C-terminal Sec residue, see Figure 4; further kinetic studies have suggested that the terminal Sec residue mediates the transfer of gold from its ligands in AF to the redox-active Cys pairs of TGR [46].

The usage of in silico molecular docking allowed to infer that AF targets the Sec in the active site of thioredoxin (Trx) glutathione reductase (SmTGR) which shuttles electrons from NADPH to Trx and to the oxidized form of glutathione (GSSG). The tight binding of the [Et_3_PAu(I)]^+^ cation to the SmTGR, forming transient interaction with the neighboring protein residues (in particular with Cys596), was found to eventuate with the transfer of gold atoms to SmTGR active site [47].

### 2.2. Au(I) Complexes with N-heterocyclic Carbene (NHC) Ligands

Metal complexes with N-heterocyclic carbene (NHC) ligands are ubiquitously utilized in chemistry due to their excellent catalytic properties [48]. In the last decade, a lot of studies unveiled the potential of metal NHC compounds in medicinal chemistry since these scaffolds can be employed for the development of powerful and efficacious metallodrugs, active against infectious diseases and cancer [21,49]. Indeed, gold complexes with N-heterocyclic carbene (NHC) ligands were found to constitute a class of promising anticancer metallodrugs with vigorous in vitro and in vivo activities. Nevertheless, their mode of action was not fully elucidated [24,50]. Possible mechanisms for initiating the cell apoptosis process are (i) direct DNA damage, (ii) mitochondrial damage via thioredoxin reductase (TrxR) inhibition and subsequent mitochondrial impairment, (iii) alteration of specific kinases, and (iv) inhibition of proteasome [51]. Nowadays, there is a consensus that the inhibition of TrxR has a crucial part in the therapeutic effect of gold complexes due to the high affinity of gold toward thiol and selenol groups [52]. Moreover, the reduction of Au(III) cations to Au(I) in the cytoplasm augments their preference to favorably bind with the thiol side chains of cysteine residues, including those involved in the coordination of other metals. In this frame, Au(I) breaks the metabolic pathways via the displacement of functional metal atoms, impeding the reduction-oxidation balance and increasing the permeability of cells, and, consequently, inducing the apoptotic response [53].

**Figure 5:**
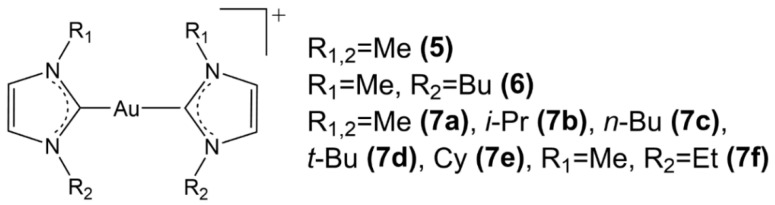
Gold(I) bis-NHC-carbene complexes (**5**, **6**, **7a**–**7f**).

Computational investigations at the DFT level on the possible usage of Au(I) bis-N-heterocyclic carbene complexes (Figure 5, structure **5**) for the attack on Cys and Sec protein residues focused on the initial steps of the carbene ligand substitution by these aminoacidic nucleophiles [54,55,56]. In ref. [55] and [56], the authors employed the capped models of neutral and deprotonated Cys and Sec and explicitly considered the presence of acidic moiety in solution, such as a neighboring acidic residue or the acidic component of the buffer milieu. It was inferred that, although able to afford the concerted protonation of the released carbene, the targeting of neutral cysteine or selenocysteine is kinetically unfavorable in physiological conditions. In contrast, the deprotonated thiolate and selenolate forms of Cys and Sec, respectively, albeit necessitating an external proton source for the protonation of the leaving carbene, tend to be more nucleophilic and, thus, more reactive. The crucial role played by the buffer in the protonation of the leaving carbene spotlights the significance assumed by the biological milieu in the mechanism of action of Au(I) bis-N-heterocyclic carbene complexes.

**Figure 6:**
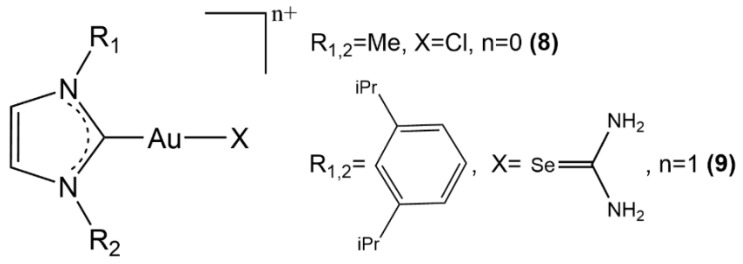
Gold(I) complexes with NHC carbenes (**8**, **9**).

Neutral N-heterocyclic carbene gold(I) compounds such as IMeAuCl with IMe = (Me_2_Imy) were recently reported as promising antitumor agents in medicinal chemistry [57]. A detailed density functional theory study was performed by aiming at the thermodynamics and kinetics of the reactions where chloride was substituted with a variety of nucleophilic protein side chains such as arginine, aspartic acid, asparagine, cysteine, glutamic acid, glutamine, histidine, lysine, methionine, selenocysteine, and the N-terminal group (Figure 6, structure **8**) [58,59]. It was shown that despite the 23.0 kcal/mol activation-free energy of chloride substitution by water, a rather accessible barrier at physiological conditions, the barriers for the analogous reactions with other nucleophiles are lower (12–20 kcal/mol), thus suggesting that the [Au(I)(NHC)Cl] complex directly attacks its biomolecular targets before the occurrence of hydrolysis. The calculated kinetic reactivity order has been established to be Sec > Lys > activated-Gln/Asn > activated-Cys > Cys > Met > Arg > N-terminal > His > Gln/Asn ≈ Glu/Asp >, well corroborated by the experimentally observed prevalent binding of a [IMeAu]^+^ fragment to Sec and Cys in thioredoxin. The overall promiscuity of this gold-based scaffold, displayed in the favorable thermodynamic and kinetic values in its reaction with the selected nucleophiles, suggests that its binding preference to a protein side-chain might be rather controlled by the accessibility (bulk exposure) of the targetable sites.

**Figure 7:**
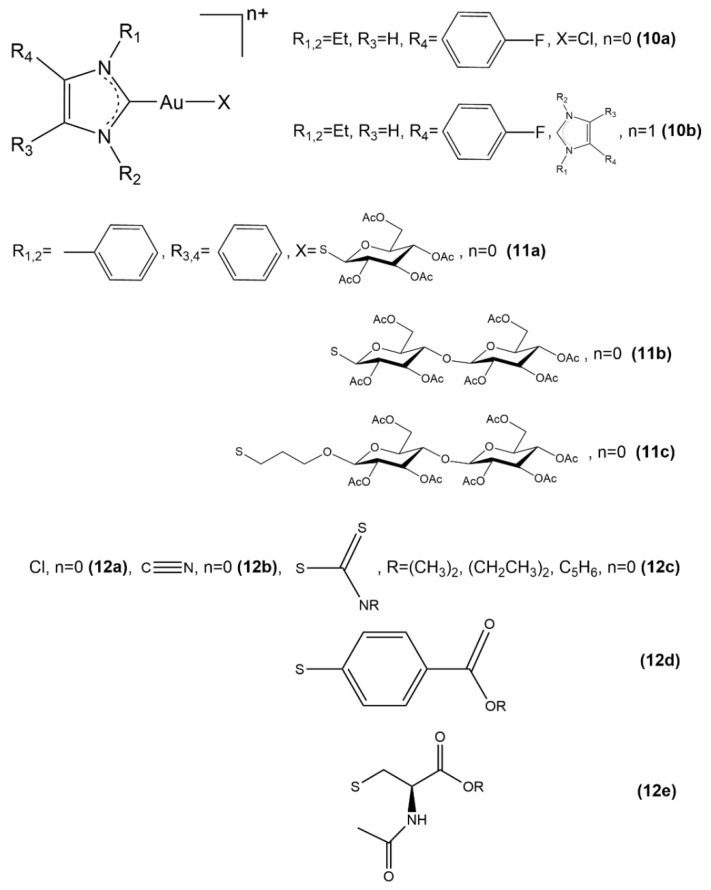
Gold(I) complexes with NHC carbenes (**10a**–**10b**, **11a**–**11c**, **12a**–**12e**).

The interaction of a set of gold-based complexes (Figure 7, structures **10a**, **10b**) with the mammalian and bacterial TrxR was computationally studied by means of docking studies focused on the binding of [(NHC)Au]^+^ and [(Et_3_P)Au]^+^ fragments with both types of TrxR isoforms [60]. Calculations displayed an exceptionally good embedding of the [(NHC)Au]^+^ fragment of compound **3** in both crystal structures, interacting more actively with the protein residues. Moreover, it was concluded that the selenocysteine in human TrxR favors the breaking up of **3,** yielding the [(NHC)Au]^+^ fragment release.

An accurate computational investigation reveals a detailed kinetic analysis of the reaction of six alkyl-substituted NHC with cysteine (Cys), which is selected for the study as an important bionucleophilic molecule (Figure 5, structures **7a**–**7f**) [54]. In this study, the first and second ligand substitution was investigated with the full characterization of their mechanism. For the first reaction step, which turned out to be rate-limiting, the calculated trend of activation enthalpies resulted in being **7a**/Me_2_ < **7f**/Me, Et < **7c**/*n*-Bu_2_ < **7b**/i-Pr_2_ < **7e**/Cy_2_ < **7d**/*t*-Bu_2_; this order correlates well with the Au-S distances observed in transition states: longer distances relate to higher values of activation enthalpies, consistently with the steric hindrance exerted by the bulky alkyl-substituted NHC ligand on the gold center. On the other hand, the higher activation barrier can be ascribed to electronic modulations originating from the enhanced electron-richness in the substituted NHC ligands, which translates in the strengthening of metal-NHC coordination. Additionally, it was demonstrated that a similar, although somewhat faded, structure––reactivity relationship characterizes the substitution of the second ligand.

Another gold(I)-carbene complex, i.e., [Au(IPr)(Seu)]PF_6_ with Seu = selenourea and IPr = 1,3-Bis(2,6-diisopropylphenyl)imidazol-2-ylidene, was found to be less potent than cisplatin (*cis*-diamminedichloroplatinum) in inhibiting the cellular growth in lung carcinoma A549, colon cancer HCT15, and breast cancer MCF7 lines (Figure 6, structure **9**) [61], and was also investigated at DFT level of theory. The usage of DFT allowed to optimize its structure and to analyze the charge distribution [61].

The NHC*-Au-thiosugar complexes based on 1,3-dibenzyl-4,5-diphenyl-imidazol-2-ylidene were investigated by experiment and DFT computations and were found to have similar strong and redox-active Au-S bond as in the structurally related auranofin (Figure 7, structures **11a**–**11c**) [62]. These complexes are soluble in the biological milieu and show good cytotoxic activity in the medium to low micromolar range, and complex **11a** shows higher activity in the low micromolar to nanomolar range against the tested cell lines [62].

Another experimental/theoretical study focused on the NHC*-Gold(I)-X complexes, where NHC* is here represented by 1,3-dibenzyl-4,5-diphenylimidazol-2-ylidene and X = chloride, cyanide, dithiocarbamates, *p*-mercaptobenzoate or *N*-acetyl-l-cysteine (Figure 7, structures **12a**–**12e**) [63]. The evaluation of the biological activities and the calculated stabilities of these complexes led to the conclusion that NHC*-Au(I)-thiolates **12c**, **12d**, **12e** are biologically more active than the complexes **12a**, **12b**, which do not possess the Au-S bond.

**Figure 8:**
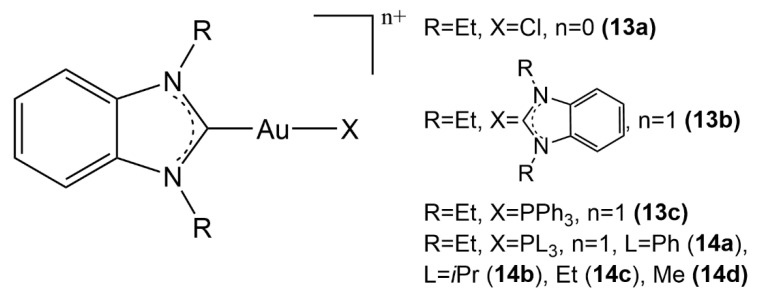
Gold(I) complexes with a 1,3-diethylbenzimidazol-2-ylidene N-heterocyclic carbene (**13a**–**13c**, **14a**–**14d**).

The joint use of experimental and computational methods allowed the evaluation of a set of gold(I) complexes with a 1,3-diethylbenzimidazol-2-ylidene N-heterocyclic carbene (NHC) ligand of the type NHC-Au-L (L = -Cl, -NHC, or -PPh_3_) as potential TrxR inhibitors (Figure 8, structures **13a**–**13c**) [64]. These metal scaffolds displayed exceptional antiproliferative properties, a strong induction of apoptosis, and enhancement of reactive oxygen species (ROS) formation, thus demonstrating their potential as new antitumor agents. The computational analyses of bond dissociation energies and charge distribution allows an explanation of the stability of these complexes. The employment of DFT calculations disclosed the differences in these three Au(I) complexes: the bond dissociation energy is the lowest in the case of chloride, thus indicating its capability to inhibit TrxR selectively and its reactivity comparable to that of auranofin. On the other hand, it was shown that the most stable among the three studied complexes are less effective in inhibiting TrxR. Indeed, the extensively delocalized positive charge in NHC-Au-NHC and NHC-Au-PPh_3_, together with the lipophilic structure of the bound ligands, make these Au(I) complexes perfect examples of delocalized lipophilic cations, which can easily permeate the hydrophobic barriers of cellular membranes, in force of the electric gradient between the cytosolic and extracellular compartments. The uptake of these two complexes, therefore, results in three times higher than for the neutral NHC-Au-Cl. Complexes **13b** and **13c** are well-balanced candidates for the role of antitumor agents.

Gold(I) NHC complexes with phosphines (Figure 8, structures **14a**–**14d**) strongly inhibit the proliferation of cancer cells, thanks to an effective intracellular uptake, as shown in a joint experimental-computational study [65]. Their activity toward TrxR and PARP-1 inhibition seems to be strongly related to the size of the alkyl and aryl groups decorating the phosphine ligand. In fact, DFT calculations demonstrated that the dissociation energy of Au-PPh_3_ bond in [Au(I)(NHC)(PPh_3_)] is lower than the Au-PR_3_ bond dissociation energy in [Au(I) (NHC)(PR_3_)] with R = alkyl, indicating a higher kinetic reactivity of this compound.

**Figure 9:**
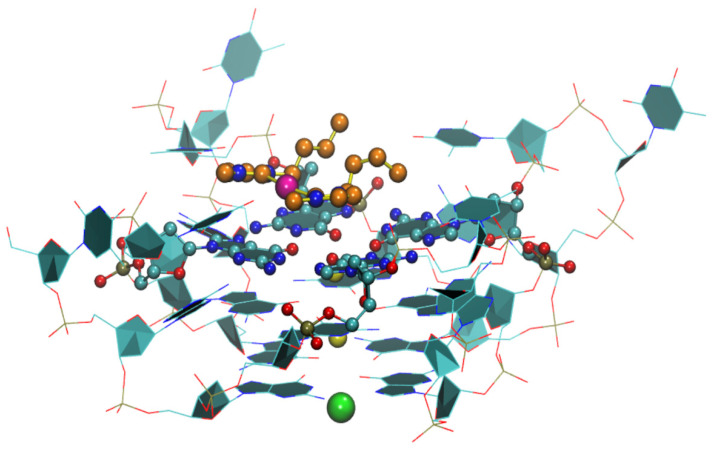
The crystal structure of a G-quadruplex unit bound to one gold complex in the [Au(NHC)_2_]^+^/Tel24 adduct [66]. The following color code has been used to identify each single atom: magenta (Au^+^),yellow (K^+^), green (Sr^2+^) and orange (C of carbene ligands). (from PDB, code 6H5R).

A different non-proteic target of Au(I)-NHC complexes are represented by the human telomeric DNA G-quadruplex (Figure 9) [66,67], representing an exciting new possibility in anticancer treatments, and its more complex computational modeling has become an intriguing challenge.

**Figure 10:**
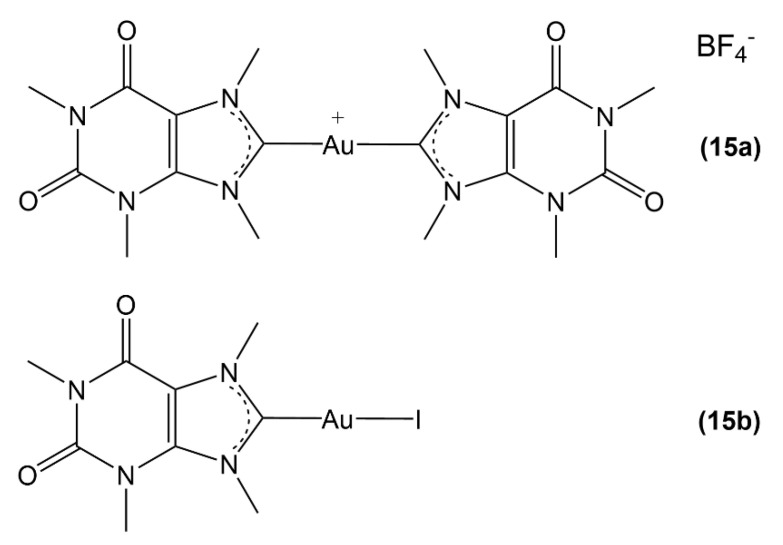
Gold(I) complexes with two (**15a**) and one (**15b**) methylcaffeine-ylidene group.

The targeting of the human telomeric (hTelo) and of a promoter sequence (C-KIT1) by Au(I)-NHC complexes was investigated via metadynamics simulations and revealed that these metal fragments might adopt several binding modes as corroborated by FRET DNA melting assays (Figure 10, structures **15a**, **15b**) [67]. The binding free energies for these Au(I)-DNA adducts were consistent with the corresponding experimental estimates. This study shows that metadynamics is a viable and promising approach for drug design, although its employment in the study of metallodrugs’ pharmacodynamics is still limited.

The biscarbene gold(I) complex [Au(1-butyl-3-methyl-2-ylidene)_2_]PF_6_ ([Au(NHC)_2_]PF_6_ hereafter) started to receive attention as a perspective anticancer metallodrug (Figure 5, structure **6**) [68] due to its remarkable stability under physiological conditions. For instance, it preserves its original structure even in the presence of glutathione (GSH). A combination of high-resolution mass spectrometry and computational DFT and QM/MM methods was used to investigate its interaction [Au(NHC)_2_]PF_6_ with the human telomeric DNA G quadruplex, see Figure 9, and it was concluded that [Au(NHC)_2_]PF_6_ binding affects the G quadruplex melting temperature and its conformation, although only marginally [66].

### 2.3. Au(I) Complexes with Other Structures

**Figure 11:**
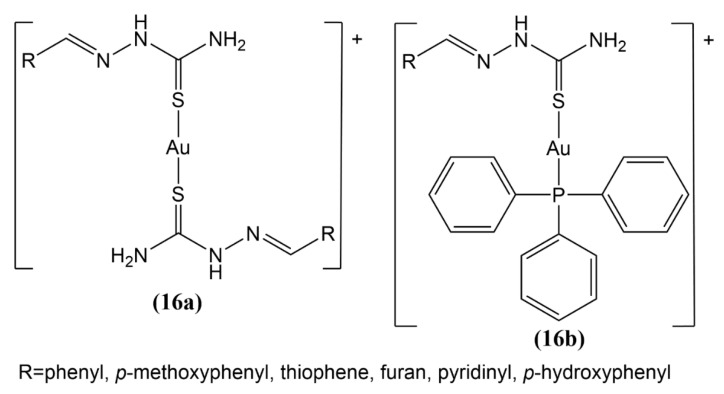
Gold(I) complexes with two (**16a**) and one (**16b**) aryl-thiosemicarbazone moieties.

The growing attention for the entering of thiosemicarbazones and their metal complexes in the field of medicinal chemistry stimulated the development of new gold(I) complexes bearing aryl-thiosemicarbazone moieties (Figure 11, structures **16a, 16b**) [26]. These complexes display a satisfactory cytotoxic activity against either tumor (B16-F10 and CT26.WT) or non-tumor cell lines (BHK-21), being even more cytotoxic in tumor cells compared to *cisplatin* and showing a high selectivity with the *phosphine* derivatives being more selective than their corresponding non-phosphine gold complexes. Docking investigations gave insights into the binding mode of these gold-based complexes at the TrxR enzyme. Both docking and experimental investigations inferred that either **16a** or **16b** complexes bearing the *p*-hydroxyphenyl substituent (Figure 11) turn out to be the most effective inhibitors of TrxR due to their size and peculiar shape that well fit the residues surrounding the anchoring site of TrxR and favor the stabilization of the metal-protein adduct.

**Figure 12:**
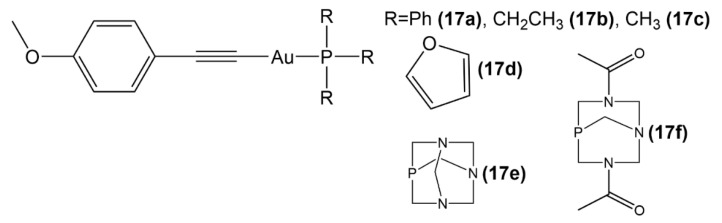
Gold(I) phosphine complexes (**17a**–**17f**).

Gold(I) complexes with alkynyl and phosphine ligands are perspective scaffolds for metallodrug design and have recently been found to exert an inhibiting activity against TrxR in a joint experimental and computational study [69]. All the six synthesized complexes (Figure 12, structures **17a**–**17f**) displayed similar cytotoxicity; however, they were not selective for tumor cells. It was noted that the cytotoxic activity was only marginally affected by the residues at phosphine; a slightly stronger cytotoxic effect was produced when the ethyl moiety (**17b**) was present. Moreover, it was indicated that the inhibition of TrxR is stronger when alkyl/phenyl residues are available (**17a**, **17b**, **17c**), while the complexes incorporating ligands with N/O heteroatoms (**17d**, **17e**, **17f**) were less effective.

**Figure 13:**
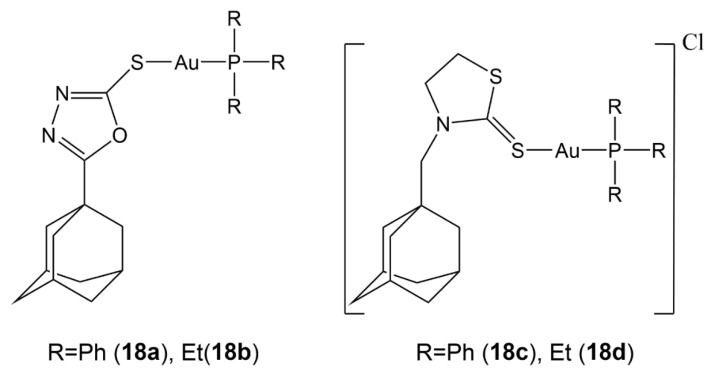
Gold(I) complexes with tertiary phosphine and 3-methyladamantane–1,3,4-oxadiazole-2-thione (**18a**, **18b**) and 5-adamantyl-1,3-thiazolidine-2-thione (**18c**, **18d**).

Novel gold(I) complexes containing tertiary phosphine and the new ligands 5-adamantyl-1,3-thiazolidine-2-thione, 3-methyladamantane–1,3,4-oxadiazole-2-thione (Figure 13, structure **18a**–**18d**) were recently synthesized [70]. Coordination of gold to the exocyclic sulfur atom was observed spectroscopically in all four complexes and was corroborated by X-ray results and quantum chemical calculations. All the four compounds were found to be cytotoxic in four different tumor cell lines, colon cancer (CT26.WT), metastatic skin melanoma (B16F10), mammary adenocarcinoma (4T1), and kidney normal cell (BHK-21). Moreover, molecular docking permitted to characterize the interaction between novel antitumor adamantane––azole gold(I) complexes and their potential target, again, thioredoxin reductase [70]. It was demonstrated that the adamantane ring is crucial for the stabilization of the metallodrug-TrxR complex before the formation of a covalent bond between gold and Sec.

## 3. Computational Studies of Au(III) Metallodrugs

Au(III) complexes have the metal center with d^8^ configuration, thus they are isostructural and isoelectronic with Pt(II) complexes, assume square planar geometry, and exhibit cytotoxic activity. It was first supposed they could show the same mode of action of platinum complexes, binding to DNA. However, contrarily to platinum(II) compounds, gold (III) analogs are more labile and, in the aqueous and reducing intracellular environment, are particularly suitable to hydrolysis and to reduction to either Au(I) or Au(0). The choice of ligand is therefore particularly important and, indeed, the stability of Au(III) compounds may be substantially enhanced by the appropriate selection of inert ligands, i.e., polydentate ligands with sulfur, oxygen, or nitrogen as donor atoms, including porphyrins, or organometallic cyclometalated scaffolds [71,72,73]. In addition, gold(III) complexes may be more easily modulated to either increase stability or decrease toxicity [74,75,76]. At variance with initial assumptions, later studies have shown that their mode of action nalogously to gold(I) compounds—is based on inhibition of sulfur and selenium-containing enzymes, such as thioredoxin reductase (TrxR), poly (ADP-ribose) polymerase 1 (PARP-1), cathepsins, aquaporins.

**Figure 14:**
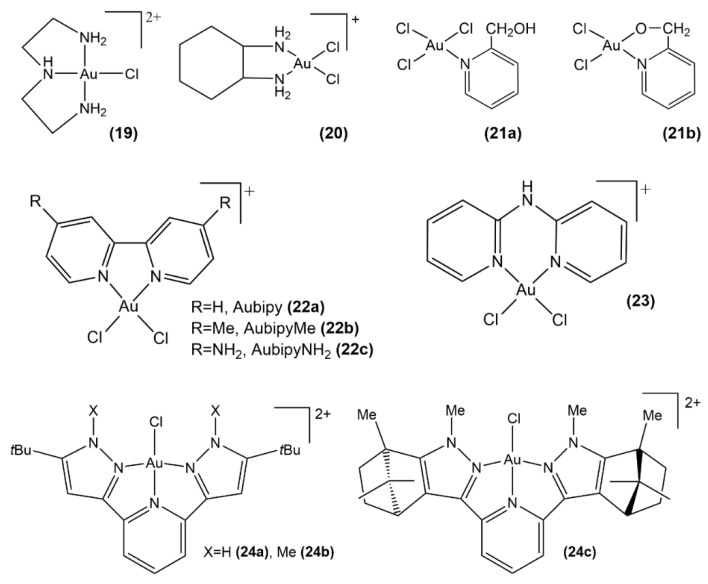
Au(III) complexes with azapentanediamine (**19**), diaminocyclohexane (**20**), pyridines (**21a**, **21b**), bipyridyl (**22a**–**22c**), dipyridin-2-ylamine (**23**), bispyrazolpyridine (**24a**, **24b**) and bisindazolpyridine (**24c**).

**Figure 15:**
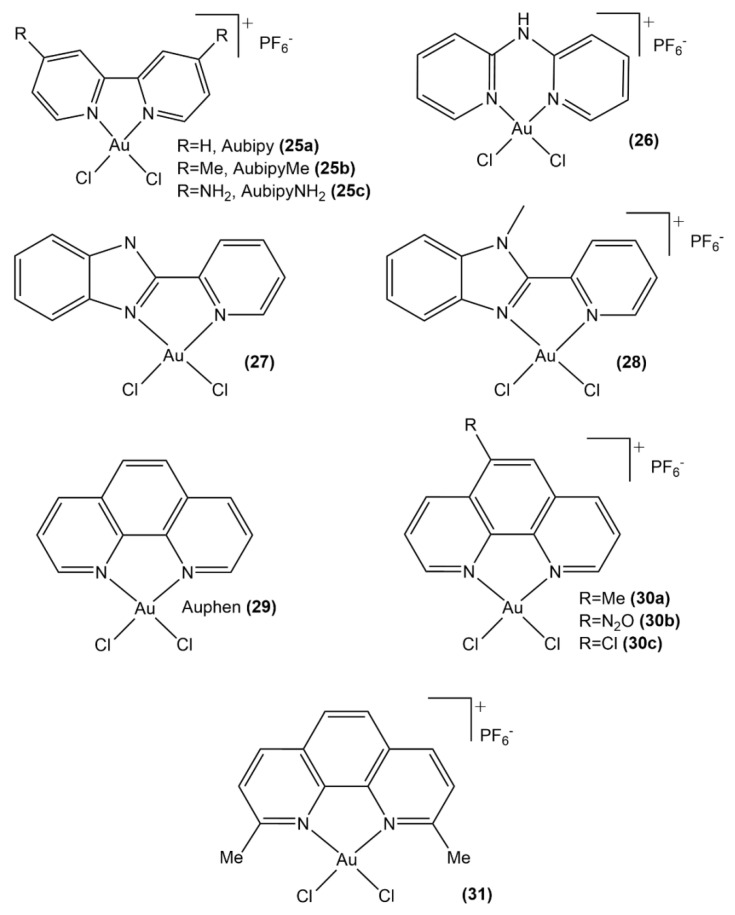
Au(III) complexes with bipyridyl (**25a**–**25c**), dipyridin-2-ylamine (**26**), pyridylbenzimidazole (**27**, **28**), and phenanthroline (**29**, **30a**–**30c**, **31**) groups.

**Figure 16:**
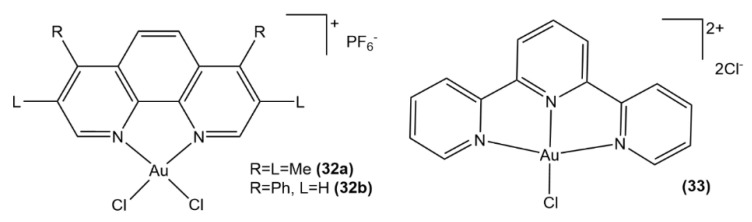
Au(III) complexes with substituted phenanthroline (**32a**, **32b**) and terpyridine (**33**).

**Figure 17:**
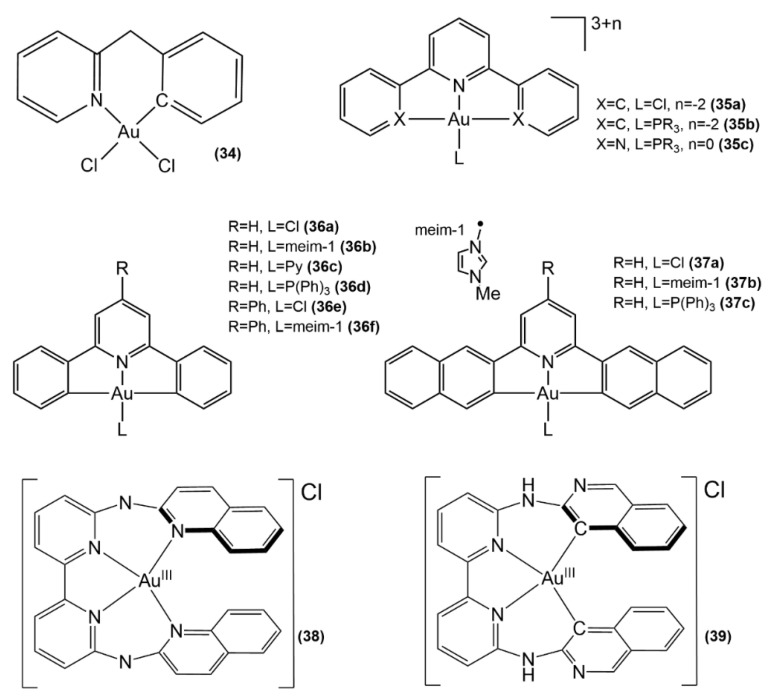
Au(III) complexes with benzylpyridine (**34**), diphenylpyridine (**35a**, **35b**), terpyridine (**35c**), diphenylpyridine (**36a**–**36f**) and naphtalene-2-yl-pyridine (**37a**–**37c**), tetrapyridyl (**38**, **39**).

**Figure 18:**
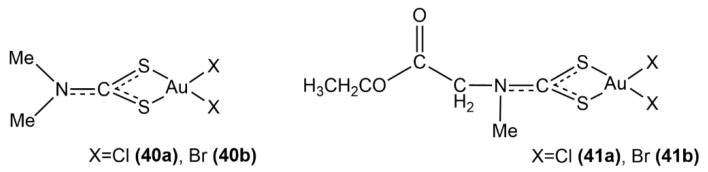
Au(III) complexes with dithiocarbamates (**40a**, **40b**, **41a**, **41b**).

**Figure 19:**
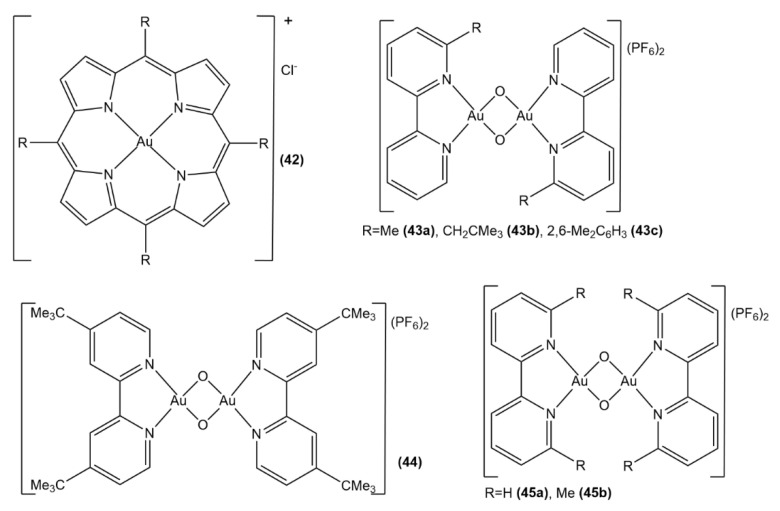
Au(III) complexes with porphyrinate (**42**) and oxo-bridged binuclear gold(III) compounds (**43a**–**43c**, **44**, **45a**, **45b**).

A number of gold(III)-based scaffolds were investigated as perspective anticancer drugs both experimentally and computationally, demonstrating effectiveness against cisplatin-resistant tumors and some of them displaying excellent cytotoxicity in vitro and in vivo against solid cancers together with low systemic toxicity [20,74]. Most of these gold(III) antitumor complexes are based on multidentate ligands, including N^N, N^N^N, C^N, C^N^N, C^N^C, porphyrins, and dithiocarbamate, see Figure 14, Figure 15, Figure 16, Figure 17, Figure 18 and Figure 19.

Several computational studies have been carried out in the last years to unveil the interaction between Au(III) center and possible biomolecular targets.

### 3.1. Interaction with Biologically Relevant Targets

#### 3.1.1. Au(III) Complexes with Chelating N Donor Ligands

The kinetics and the mechanism of the substitution reactions between the mono-functional Au(III) complexes, [Au(dien)Cl]^2+^ and [Au(terpy)Cl]^2+^ (dien = 3-azapentane-1,5-diamine, terpy = 2,2′;6′,2″-terpyridine) (Figure 14, structure **19** and **33**) and bi-functional Au(III) complexes, [Au(bipy)Cl_2_]^+^ and [Au(dach)Cl_2_]^+^ (bipy = 2.2′-bipyridine, dach = (1*R*,2*R*)-1,2-diaminocyclohexane) (Figure 14, structures **22a** and **20**) and biologically relevant targets such as L-histidine (L-His), inosine (Ino), inosine-5′-monophosphate (5′-IMP) and guanosine-5′-monophosphate (5′-GMP), were comprehensively investigated [77], revealing an intriguing result in that the reactions of mono-functional complexes were shown to be more rapid than the reactions of bi-functional complexes. DFT calculations corroborated the higher reactivity of [Au(terpy)Cl]^2+^ compared to [Au(dien)Cl]^2+^, with activation energy for the chloride exchange in the terpy complex a half lower than the dien complex. On the other hand, the bi-functional [Au(bipy)Cl_2_]^+^ complex disclosed an augmented reactivity, compared to [Au(dach)Cl_2_]^+^. The reactivity order with the biomolecular targets is similar for all the above Au(III) complexes, hence L-His > 5′-GMP > 5′-IMP > Ino. Quantum chemical calculations [77] indicated that the ligand exchange in most of these complexes takes place via an associative mechanism, whereas the imidazole attack on [Au(terpy)Cl]^2+^ and [Au(dien)Cl]^2+^ is better described with an associative interchange mechanism. There is a pronounced correspondence between the reactivity of the studied metal scaffolds toward biological targets and their structural and electronic properties. Moreover, this study elucidated the coordinative binding of gold(III) complexes to 5′-GMP, a DNA nucleophile site of recognized importance since its binding is presumed to account for the anticancer activity.

A recent DFT study focused on the interaction of pyridine gold (III) complexes Au(Hpm)Cl_3_ and Au(pm)Cl_2_ (Hpm = 2-pyridylmethanol) with cysteine and purine bases (Figure 14, structure **21a**, **21b**) [78]. It was demonstrated that hydrogen bonding and proton transfer are indispensable for stabilizing the produced complexes and for diminishing the activation energy. Interestingly, guanine displayed a more marked reactivity with gold (III) complexes in comparison with both adenine and cysteine. Moreover, the biological milieu in which the reaction takes place greatly affects the reaction due to the possibility of the formation of zwitterions. It was shown that the binding of studied gold(III)-based complexes to oxygens of Cys was kinetically more selective than the analogous metalation of cysteine sulfur. In contrast, both guanine and adenine demonstrate complete independence on the substrate types because their reactivity was found to be only marginally affected by the solvent.

Interactions of novel gold(III) complexes with essential biomolecular targets and DNA/BSA were characterized by various experimental methods and by theoretical calculations (Figure 14, structure **24a**–**24c**) [79]. Complexes **24a**–**24c** showed a good affinity toward different biomolecules, such as Guo, 5′-GMP, and DNA, indicating the capability of these gold(III) complexes to target DNA/BSA. Indeed, the UV-Vis and fluorescence measurements corroborated their interactions with DNA characterized by high binding constants in the range 10^3^–10^4^ (M^−1^), whereas the calculated constants for the binding of BSA were found to vary between 10^4^ and 10^5^ (M^−1^). Calculations confirmed the experimental data, and molecular docking studies were used to detail the anchoring of these metal complexes at DNA/BSA. The anticancer potential of the studied Au(III) complexes was supported by cytotoxic activity assays against various cancer cell lines.

Aquaporins (AQPs) are the cellular membrane channels that transfer water, glycerol, and other smaller chemical species, such as hydrogen peroxide, under the influence of osmotic gradients [80]. The AQPs are found in a myriad of human cell types and participate in the control of the urine concentration, metabolism of fat, wound healing, and hydration of skin [81]. Moreover, various isoforms of these transmembrane channels are overexpressed in human tumors, being connected to various types, grades, and stages of cancers [82]. This variability serves as the basis for the identification of AQPs as amenable targets of anticancer drugs. Up to date, no organic inhibitor of AQP was reported as a satisfactory choice for the clinical development of antitumor therapeutic agents. Nevertheless, a set of recent studies on Au(III) metal complexes indicates their unique abilities in providing a selective targeting of the aquaglyceroporins AQP3 [83].

QM/MM and DFT calculations were successfully used for the investigation of the most likely molecular scenario through which the gold(III) complexes with nitrogen donor ligands, such as 1,10-phenatroline, 2,2′-bipyridine, 4,4′-dimethyl-2,2′-bipyridine, 4,4′-diamino-2,2′-bipyridine, and 2,2′;6′,2″-terpyridine (Figure 14, structures **22a**–**22c**, Figure 15, structure **29**, Figure 16, structure **33**), interact with aquaporin AQP3 [84]. This study supports the previously developed model of inhibition of AQP3 by gold(III)-complexes, in which the metal tightly coordinates the thiol side chain of Cys40, which is located near the selectivity filter of this protein channel and induces the channel blockage. It was found that the considered gold(III) complexes manifest selectivity toward AQP3 over AQP1. The identification of the Cys40 of AQP3, proximal to the SF domain, as the most plausible Au(III) coordination site, was initially gained via homology modeling calculations [84] and, subsequently, corroborated via site-directed mutagenesis experiments [85] (vide infra).

The bipyridyl gold(III) complexes Auphen ([Au(phen)Cl_2_]Cl phen = phenantroline) and Aubipy ([Au(bpy)Cl_2_]PF_6_ bpy = bipyridine) have a unique ability to selectively cause the blockage of AQP3. Classical MD simulations produced a comprehensive picture of Aubipy interacting with AQP3 by assessing the structural response of AQP3 to either covalent or non-covalent binding of Aubipy (Figure 14, structure **22a**) [86]. The comparison of the unbound, and either covalently—at Cys40 or non-covalently bound Au(III)-AQP3 adducts in terms of their structure and dynamics led to conclude that coordination of gold(III)-based complex induces conformational changes within the AQP3 channel that eventually lead to the impairment of its functionality. Such structural modifications of the AQP3 extracellular pore resulted in a decline of the water molecules flux, as assessed by the analysis of the channel radius and water density along the transmembrane. The most evident structural alteration of the pore ascribed to the binding of the gold fragment is represented by its shrinkage that prevents or limits the fluxing of water along this protein channel. The computational analyses allow gaining insights into the design of AQP3 inhibitors with ameliorated characteristics, in particular, via the insertion of substituents of the aromatic ligands to stabilize and/or reinforce the hydrophobic and π-stacking interactions involved in the Au(III)-AQP3 binding.

Other gold(III)-based complexes (Figure 15, structures **26**–**29**, Figure 17, structure **34**) [85] were studied by stopped-flow spectroscopy and molecular dynamics combined with DFT. They support the Cys40 as the main target of gold metallodrugs in AQP3 and its blockage induction causing disruption of glycerol and water permeation, as the main *modus operandi* of the studied metal complexes. Interestingly, the cationic compound **28** was found to be the most effective inhibitor of glycerol permeation, whereas complex **27** with a similar ligand was only marginally active. The computational study allowed an explanation of this aspect with the facile complexation of the cationic complex **28** [Au(PbImMe)Cl_2_]^+^ by cysteinato residues, whereas the neutral complexes [Au(pyb)Cl_2_] **34** and [Au(PbIm)Cl_2_] **27** are less prone to be complexed due to less favorable electrostatics.

Auphen was found to be an exceptionally effective inhibitor of AQP3 while being a rather weak inhibitor of AQP1, thus displaying a remarkable degree of selectivity (Figure 15, structure **29**) [87]. Computational studies joint with experimental insights provided further evidence that its inhibitive properties originate from the Au(III) propensity toward interaction with sulfhydryl moieties of proteins, such as the thiol group of Cys40 of AQP3. Additionally, molecular docking corroborated the selectivity of Auphen to AQP3 with respect to AQP1, which, together with its high solubility in water and a proven inhibitory effect even at low concentrations (in the nanomolar range), makes this metallodrug a perfect candidate for future in vivo studies.

A set of six Au(III) complexes with substituted 1,10-phenanthroline ligands (Figure 15, structures **30a**–**30c**, **31**, Figure 16, structures **32a**, **32b**), which inhibit the water and glycerol channel AQP3, were synthesized and studied in a joint experimental-computational study [88]. Both DFT computations and UV-Vis spectrophotometry data showed that these complexes do not undergo hydrolysis via the exchange of chloride ligands with water or hydroxyl anion, which makes them different from Au(III) bipyridyl complexes. Their cytotoxic activity against A549 human lung cancer cells is a sign of their ability to inhibit cell proliferation in vitro, whereas these metal-based scaffolds strongly disrupt the glycerol penetration in human red blood cells (hRBC) by means of AQP3 inhibition. Their affinity for thiols is more pronounced compared to Aubipy ([Au(bipy)Cl_2_]PF_6_, bipy = 2,2′-bipyridine), as shown by a QM study of the ligand exchange with methanethiol modeling the Cys40 side chain of AQP3. Moreover, a multilayered computational approach incorporating QM, molecular dynamics MD, hybrid QM/MM methodologies, as well as the analyses of atoms in molecules (AIM) and natural bond orbitals (NBO), was employed for investigating both coordinative and non-covalent binding of these Au(III) complexes to the AQP3 channel, characterizing it as result of complex protein conformational changes taking place concomitantly with the coordinative gold binding, leading to the pore blockage. These kinds of computations are an indispensable basis for the future design of isoform-selective AQP inhibitors since they shed light on the significance of non-coordinative adducts in fine-tuning the AQP3 inhibition.

#### 3.1.2. Au(III) Complexes with Cyclometalated Ligands

The high redox stability is one of the crucial features of cyclometalated Au(III) compounds characterized by the presence of at least one metal-carbon σ bond [89,90]. However, the decrease in the formal positive charge of in Au(III) compounds gained by cyclometalation substantially alters its biological properties, in particular its reactivity with serum proteins and its uptake in cells [91]. The majority of cyclometalated Au(III) complexes with significant cytotoxicity include a bipyridine or terpyridine ligand and monodentate ligand(s) [92,93]. Cyclometalated Au(III) complexes with tetradentate ligands are instead uncommon in a biological milieu [91,94]. The cyclometalated complexes **35a**–**35c**, **36a**–**36f**, **37a**–**37c** (Figure 17) feature a remarkable redox activity, giving rise to peculiar pathways of protein targeting. Theoretical investigations on the structure and reactivity of such complexes [95,96,97,98] are reported below (vide infra, Section 3.2. Redox stability).

Recently, the anticancer properties of two bis-cyclometalated gold(III) complexes by *N*6,*N*6′-di(quinolin-2-yl)-[2,2′-bipyridine]-6,6′-diamine (Figure 17, structure **38**) and *N*6,*N*6′-di(isoquinolin-3-yl)-[2,2′-bipyridine]-6,6′-diamine (structure **39**) were studied in a joint experimental and computational study [99]. Complex **39** was found to be stable in the presence of biological thiols, whereas **38** produced the metastable Au(I) species after reduction in a millimolar concentration of glutathione, thus releasing the TrxR-inhibiting Au^+^ ions. The redox stability of **39** decreases significantly its ability to inhibit thioredoxin TrxR; however, unlike complex **38**, it aggregates into nanoparticles in a biological medium, thus causing a more efficacious gold uptake by cells. Moreover, the lesser promiscuity of **39** makes it ten times more cytotoxic (with respect to cisplatin and **38**) to human cancer cells (A549, A431, A375, and MCF7) than to noncancerous cells (MRC5). DFT calculations elucidated the contrasting mechanisms of action of both complexes: **38** was found to produce the TrxR-inhibiting Au^+^ ions and toxic tetrapyridyl moieties, which were found to target the hERG potassium channel responsible for the generation of cardiac toxicity in vivo, whereas **39** is characterized by a high gold cellular uptake, nuclear DNA damage, and interaction with hERG.

#### 3.1.3. Au(III) Complexes with Cyclometalated Ligands

The reactivity and selectivity of gold(III) complexes with dithiocarbamate ligands were investigated computationally (Figure 18, structures **40a**, **40b**, **41a**, **41b**) [100], revealing the importance of interatomic sulfur-halogen coordination in the stabilization of the metallic scaffold. Reactivity and selectivity toward TrxR were found to be related to the presence of charge transfer effects––either interatomic, within the gold(III) complex, or intermolecular, between the metal complex and TrxR enzyme. Both in vitro analyses and binding free energy estimates have identified complex **40a** as the most reactive towards the TrxR targeting.

Another way to stabilize gold(III) ions in the development of physiologically stable antitumor gold(III) complexes is represented by the tight chelation with porphyrinato ligands [101,102]. A set of gold(III) tetraarylporphyrins with porphyrinato ligands containing different peripheral substituents on the *meso*-aryl rings were recently synthesized, and their biological activity was investigated both in silico and in vitro (Figure 19, structure **42**) [103]. A combination of docking and protein expression analysis experiments indicated the anti-apoptotic protein bcl-2 as the biomolecular target for these gold(III)-porphyrin complexes, the inhibition of which leads to cellular apoptosis. The versatility of the porphyrin structure functionalization together with the highly stable chelation of gold(III) are the features that make this scaffold promising for the further development of physiologically stable anticancer gold(III) complexes.

It was recently shown that the oxo-bridged binuclear gold(III) compounds, [Au_2_(μ-O)_2_(N^N)_2_](PF_6_)_2_, where N^N is 2,2′-bipyridine or a substituted 2,2′-bipyridine, possess substantial stability in physiological conditions and reveal cytotoxic activity against several cell lines of human tumors [104]. Selected complexes, such as [Au_2_(μ-O)_2_(bipy)_2_](PF_6_)_2_, *cis*-[Au_2_(μ-O)_2_(6-Mebipy)_2_](PF_6_)_2_, *trans-*[Au_2_(μ-O)_2_(6-oXylbipy)_2_](PF_6_)_2_, and [Au_2_(μ-O)_2_(6,6′-Me_2_bipy)_2_](PF_6_)_2_, were crystallographically and computationally characterized. Interestingly, these complexes disclose a correlation between their oxidizing power and their antiproliferative activity (Figure 19, structure **43a**–**43c**, **44**, **45a**, **45b**) [105]. Indeed, the most oxidant species [Au_2_(μ-O)_2_(6,6′-Me_2_bipy)_2_](PF_6_)_2_ was found to be also the most reactive towards protein models as well as the most cytotoxic.

### 3.2. Redox Stability

The mechanism of action of most Au(III) complexes is a multistep process, in which the exchange of ligands between the metal scaffold and the cellular nucleophiles takes place after the permeation of the complex in the cytoplasm [106]. Additionally, Au(III) may reduce to Au(I) under physiological conditions, thus opening a route for further ligand substitution reactions of Au(I) compound [73]. In the design of effective Au(III) metallodrugs, the formulation of computational protocols for an accurate prediction of reduction potentials may be particularly insightful, although such estimates, expectedly quite sensitive to the energy contributing terms (mostly nuclear repulsion, electronic energy, thermal correction, and solvation energy) can only be afforded at a high level of theory. The DFT studies reported in the present Section demonstrate the effectiveness of quantum chemical approaches for the determination of redox potential for bioinorganic systems incorporating gold-based scaffolds.

**Scheme 1:**
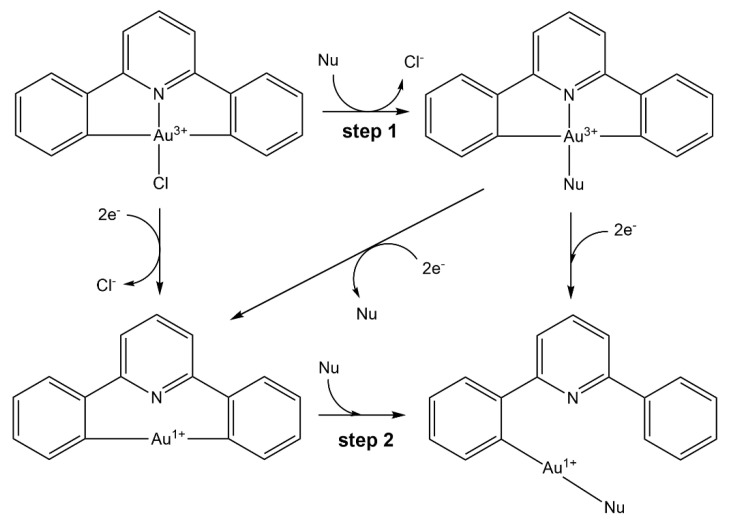
The lowest energy reaction path for a typical Au(III)-based complex is composed of two consecutive steps: (1) substitution of chloride by the nucleophile and (2) reduction of the resulting Au(III) complex to the corresponding Au(I) derivative with the opening of the chelate ring.

Distinctive features of Au(III) anticancer complexes, which make them unique, are their redox instability and the high affinity of gold towards cellular nucleophilic targets. An exceptionally detailed computational study focused on the interaction of [Au(C^N^C)Cl] probe complex (C^N^C = 2,6-diphenylpyridine) with water and the biomolecular targets represented by simplified molecular models CH_3_SH/CH_3_S^−^, CH_3_Se^−^, and 4-methylimidazole (Figure 17, structure **35a**, Scheme 1) [95]. DFT calculations permitted to conclude that the lowest energy reaction path is composed of two consecutive processes: (a) the substitution of chloride by the nucleophile and (b) the reduction of the resulting Au(III) complex to the corresponding Au(I) derivative with the opening of the chelate ring. This study disentangles the reaction mechanism of Au(III) complex and its biomolecular targets and elucidates the processes behind the therapeutic effects.

The biological milieu also has a crucial effect on the reactivity of the Au^III^-C^N^C antitumor complexes (Figure 17, structure **35a**), as shown in a recent computational study [96]. As mentioned above, the activation of Au(III) complexes can be described as occurring in two steps. In this case, the reduction of Au(III) to Au(I) takes place only after the initial ligand exchange reaction has led the metal fragment to bind at the cellular cysteines in the active site of TrxR. Simplified models were employed in order to imitate the structure of the enzyme characterized by the presence of the C-terminus near the active site of TrxR and in close proximity to the gold fragment bound to Cys. The reduction potential increases with the ligand substitution by Cys in the active site of TrxR, thus, demonstrating that the redox stability of this prototypical Au(III) complex is substantially affected by the nature of the auxiliary ligand, giving a further understanding of how to further fine-tune Au(III) metal scaffolds.

One of the strategies adopted to control the reactivity of Au(III)-based antitumor complexes towards their biomolecular targets is the use of appropriate ligands. Due to the exceptional stability and lipophilicity, tertiary phosphines are widely utilized in the design of Au(III)-based metallodrugs [97]. In order to investigate the effects of various phosphines onto the redox stability of [Au(III)(C^N^C)PR_3_]^+^ (Figure 17, structure **35b**) (C^N^C = 2,6-diphenylpyridine, PR_3_ = tertiary phosphine) and [Au(III)(N^N^N)PR_3_]^3+^ (N^N^N = 2,2′:6′,2″-terpyridine) complexes (structure **35c**), a computational study was carried out to estimate the standard reduction potential ε^o^ for Au^3+^/Au^+^ [97]. It was found that εo values were negative for structures **35b** and positive for structures **35c** with varying spreads of 829 and 507 mV, respectively. Interestingly, it was inferred that the buried volume of phosphines, a descriptor proposed in ref. [107], is the major parameter for predicting the stability of complex. The electron-donating ability of phosphines has a greater impact on the redox stability of complexes **35c** than complexes **35b** since the phosphine affects both Au(III) and Au(I) forms of **35c** where the steric effects turn out to be stronger in Au(I) reduced complexes. These considerations are of utmost importance for the prediction of the redox stability of Au(III) complexes, characterizing their reactivity with biomolecular targets.

A source of instability for anticancer Au(III) complexes under physiological conditions originate from their high Au^3+^/Au^+^ reduction potential. It is therefore essential to take into account the redox potential in the design of the Au(III) complexes with overall higher stability and improved biological activity. The standard reduction potential of nine Au(III) organometallic complexes of the type [Au^3+^(R-C^N^C)L]_n_ (Figure 17, structures **36a**–**36f**, **37a**–**37c**) was computed by the density functional theory approach by producing estimates with the absolute error lower than 170 mV [98]. It was found that the range-corrected functional CAM-B3LYP with the SMD solvation model performed better than B3LYP and ωB97XD and B3LYP, with an absolute error of 82 mV in comparison to the ferrocene/ferrocenium reference electrode potential. Moreover, the employment of variable-temperature H-atom addition/abstraction method for the redox couples with non-equivalent charges resulted in a crucial enhancement of the accuracy in the computation of reduction potential, yielding the mean absolute error of only 87 mV without any scaling procedure, being 1.65-fold lower than 144 mV produced by standard procedure.

## 4. Challenges, Strengths, and Limitations of Current Computational Approaches

Various computational tools utilized in the studies of the reactivity of gold-based metallodrugs with their biomolecular targets––ab initio quantum chemistry, density functional theory, hybrid quantum mechanics/molecular mechanics (QM/MM), classical molecular dynamics (MD), and molecular docking approaches have their advantages and limitations. Quantum chemistry methods are employed for describing the complex chemistry behind the binding of metal scaffolds with biological targets, whereas the conformational variability and the steric effects are unaccounted for by these methods due to their poor scalability. On the other hand, classical approaches are used for the description of macromolecules and of their large conformational space, yet they lack the accuracy of quantum chemistry. The hybrid QM/MM methods share the strengths and weaknesses of both accurate quantum chemical and scalable classical approaches. The key point is the appropriate selection of the methodology, depending on the size of the studied system and the computational goals.

In most cases contemplated in this review, the computational investigation of the anticancer properties of either Au(I) or Au(III) stems from the identification of the chemical events culminating in the metalation of relevant biological targets. For instance, a myriad of the mononuclear Au(I) complexes [39,40,41,42,43,52,54,55,56,57] were mainly investigated for their capability to exchange labile ligands with suitable protein nucleophilic sites, such as Sec, Cys, His side chains, or other accessible coordinative groups. The support of accurate geometry optimization, typically achieved by means of DFT approach, has allowed in several instances to parallel the experimental data and to further rationalize the trend of activity for Au(I)-based metallodrugs [26,43,45,62,63,64,65,67,69]. To this purpose, the possibility to perform an accurate benchmarking of the level of theory by a thorough examination of basis set/XC-functional combinations is particularly valuable. However, to the best of our knowledge, most computational studies devoted to the structural characterization of Au(I)-metallodrugs reside on the choice of one DFT level of theory without the support of preliminary benchmarking calculations. Among the targets recognized by Au(I)-metallodrugs, the TrxR selenocysteine is undoubtedly identified as the most promising one for its potential role in cancer development [92,93]. Many computational studies [26,39,46,60,70] have therefore been specifically addressed to the metalation of this protein. DFT approaches have been valuably applied to the investigation of the thermodynamics and kinetics of the reaction between Au(I)-metallodrugs and Sec [39,40,41,42,43,52,54,55,56,57]. A challenging aspect that we believe important in the computational study of Au(I)-metallodrugs, and their TrxR targeting, is represented by the proton exchange events concomitant to the metalation process. For example, (NHC)_2_Au(I) complexes react by exchanging one NHC ligand with the target nucleophilic site; however, the concomitant protonation of the leaving ligand is the thermodynamic driving force. The correct modeling of such a parallel prototropic step is fundamental to correctly assess the thermodynamics of such an NHC/Sec exchange. These considerations have driven the DFT investigation performed by Re et al. [55,56] that hypothesized the bulk assistance to the NHC ligand exchange in the reaction between (NHC)_2_Au(I) and plausible protein targets, and that led to a significant improvement of the computational prediction of thermodynamics compared to previously reported studies [54]. The correct treatment of the bulk effects is a crucial aspect in the DFT investigation of Au(I)- or Au(III)-metallodrugs. In the latter case, based on the harder character of the Au(III) metal center, the computational study of the possible hydrolytic processes is fundamental to identifying the active species of the metallodrug. For instance, the Aubipy targeting of the Cys40 of AQP3 hypothesized [87] and subsequently confirmed by Casini et al. [84,85] has been computationally analyzed by considering the viability of preliminary aquation steps [86,88]. From a computational point of view, the assessment of the thermodynamics of these bulk processes is particularly challenging because affected by substantial entropy contributions that cannot be easily estimated by routine DFT approaches. In these instances, the use of thermodynamic cycles, allowing the admixture of experimental and theoretical estimates, has allowed establishing that Aubipy and its analogs react with the Cys40 of AQP3 in their chloro-hydroxo form, thus behaving as monofunctional metalation agents [86,88]. Another relevant aspect in the computational investigation of Au(I) and Au(III) metallodrugs is represented by the viability of either Au(III)→Au(I) or Au(I)→Au(0) reduction processes. Indeed, due to the possible reductive elimination affecting either Au(I) or Au(III) scaffolds, many metallodrugs are coordinated by ligands that provide for redox stabilization [95,99,100,101,102,103]. DFT and, more generally, ab initio approaches can be potentially employed to probe the redox stability of gold metallodrugs, even before and after the target metalation has occurred. However, the computational estimate of the reduction potential of these complexes is particularly challenging and requires a careful choice of the exchange-correlation potential and solvation model. On the one hand, a fully theoretical assessment of reduction potentials would generally involve the investigation of reduction half-reactions requiring the explicit handling of elusive species like free electrons. On the other, significant relativistic effects in the core electrons of gold are expected to be a major determinant in the theoretical estimate of Au(III)→Au(I) or Au(I)→Au(0) reduction potentials. In this respect, the routine employment of pseudopotentials for the treatment of relativistic effects may not be adequate in the investigation of the redox stability of gold metallodrugs.

The most common limitation to the tout court application of full DFT approaches in the investigation of gold metallodrugs is ascribed to the effectiveness in the use of reduced models to investigate the metal target. Indeed, when a metallodrug is expected to bind at biological macromolecules, such as proteins or nucleic acids, the structure of the target itself may have a marked influence on the metalation process, for example, by facilitating/limiting the accessibility of the metal scaffold, or by exerting a chemical control on the metalation [9,108,109]. In these cases, QM/MM approaches may be preferred to the full DFT treatment because they allow treating the steric and electrostatic influence of the target structure on the metalation process. For example, the formation of non-covalent adducts between Aubipy-based metallodrugs and AQP3 has been ascertained by QM/MM calculations, which have evidenced the importance of the metal center in the formation of weak interactions with AQP3 residues [86,88].

Classical mechanics-based, MD and docking approaches can also give useful information on the binding site in large biomolecular targets, such as proteins or DNA, provided a careful parametrization of the metal force field is employed. Indeed, the crucial drawback of classical MD simulation of bioinorganic systems is the predominant unavailability of parametrization of the metal ion in the most commonly used empirical force fields. For this purpose, the use of MD and docking approaches to investigate the interaction between gold complexes and biomolecular target requires precursory quantum chemical studies to parametrize the gold scaffold and obtain appropriate parameters for their successive employment in the empirical force field. For instance, as discussed above, MD simulations allowed a comprehensive picture of Aubipy interacting with AQP3 by assessing the structural response of AQP3 to either covalent or non-covalent binding of Aubipy [86]. In a recent study, stopped-flow spectroscopy and molecular dynamics combined with DFT allowed to identify Cys40 as the main target of gold metallodrugs **26**–**29** and **34** in AQP3 and the induced mechanism of disruption of glycerol and water permeation, shedding light on the *modus operandi* of these metal complexes [85].

## 5. Conclusions

The success of cisplatin as an anticancer drug has led to numerous studies focused on the design of novel transition metal compounds. Gold-based complexes are one of the main directions in this research due to the strong affinity of Au(I) to cysteine and selenocysteine side chains of the protein residues. This targeting allows the inhibition of sulfur- and selenium-containing enzymes such as glutathione reductases, glutathione peroxidase, glutathione-S-transferase, cysteine protease, thioredoxin reductase (TrxR), and poly (ADP-ribose) polymerase 1 (PARP-1).

For the delivery of Au(I) into the tumor cells, it is necessary to decorate it with ligands that fine-tune its reactivity and convey these gold compounds to their targets, preventing them from participating in chemical side reactions beforehand. One of the possible routes is the use of specific ligands at the linear di-coordinate Au(I) metal center, such as phosphine, thiosugar, N-heterocyclic carbene, alkynyl, and thiosemicarbazone. These drawbacks are even more pronounced for Au(III) complexes which are labile and prone to reduction to either Au(I) or Au(0) in the aqueous or physiological milieu, thus requiring an appropriate selection of inert ligands which produce the stabilizing effect on the metal core. Usually, polydentate ligands with sulfur, oxygen, or nitrogen as donor atoms, including porphyrins or organometallic cyclometalated scaffolds, are employed. A balanced choice allows for an accurate adjustment of stability and toxicity, making gold(III)-based scaffolds perspective anticancer drugs.

The role of computational chemistry in the quest for new metallodrugs has grown due to the increasing efficacy of the methods and to the parallel increase in the hardware capabilities. Ab initio and DFT methods are nowadays the best choices for describing the mode of action of the metal-based complexes characterized by the promiscuity of metal center, its multitargeting modus operandi, the widespread necessity for activation via hydrolysis or solvolysis, and the substitution character of the metallodrug reactions with their targets.

This review is focused on the recent computational studies of Au(I) and Au(III) antitumor compounds, this topic being of utmost importance for the design of novel gold-based metallodrugs. Considering the described successes of computational modeling of gold-based complexes, we highlight the importance of in silico studies of developed metal compounds as a crucial phase even before in vivo and in vitro testing. The comprehension of the mechanistic aspects underlying the metallodrug activation, target selection, target binding is important for the design of metal-based compounds and their further structural improvement, as well as for the reduction in their toxicity effects and the enhancing of targeted drug delivery, allowing a rational drug design.
